# Influence of CAR T-cell therapy associated complications

**DOI:** 10.3389/fonc.2025.1494986

**Published:** 2025-02-20

**Authors:** Mohammad Mussab Umair, Xun Lai, YuanBo Xue, Hong Yao

**Affiliations:** ^1^ Cancer Biotherapy Center & Cancer Research Institute, Peking University Cancer Hospital Yunnan, Yunnan Cancer Hospital, The Third Affiliated Hospital of Kunming Medical University, Kunming, China; ^2^ Department of Hematology, Peking University Cancer Hospital Yunnan, Yunnan Cancer Hospital, The Third Affiliated Hospital of Kunming Medical University, Kunming, China; ^3^ Cancer Biotherapy Center, Peking University Cancer Hospital Yunnan, Yunnan Cancer Hospital, The Third Affiliated Hospital of Kunming Medical University, Kunming, China

**Keywords:** CAR T-cell, immunotherapy, adverse of CAR-T, CRS, neurologic toxicities

## Abstract

Since the introduction of chimeric antigen receptor (CAR) T-cell therapy, it has elicited an immense response in both targeted and residual cancers. Its clinical efficacy is often accompanied by a group of side effects that may become serious because of factors such as tumor burden, the extent of lymphodepletion, and the type of co-stimulus. It is also crucial to know the common toxicities associated with CAR T-cell therapy, including cytokine release syndrome (CRS), immune effector cell-associated neurotoxicity syndrome (ICANS), cardiotoxicity, metabolic disorders, pulmonary toxicity, macrophage activation syndrome (MAS), prolonged cytopenia, coagulation disorders, and potential off-target effects on various organs. If not well managed, these can be fatal. However, knowledge about molecular pathways, calcineurin inhibitors, IL-6 receptor antagonists, steroids, suppression of nitric oxide synthase, various therapeutic approaches, and other recent advances have been developed to mitigate the fatal results of various short-term and chronic adverse events related to CAR T-cell therapy. This study provides a comprehensive perspective on contemporary management strategies and presumed causative processes of CAR T-cell-related adverse effects, albeit with several limitations. When CAR T-cell complications, costs, and challenges of toxicity management are properly considered, the CAR T-cell therapy of the future will include a number of toxicity-escaping options.

## 1 Introduction

**Figure 1:**
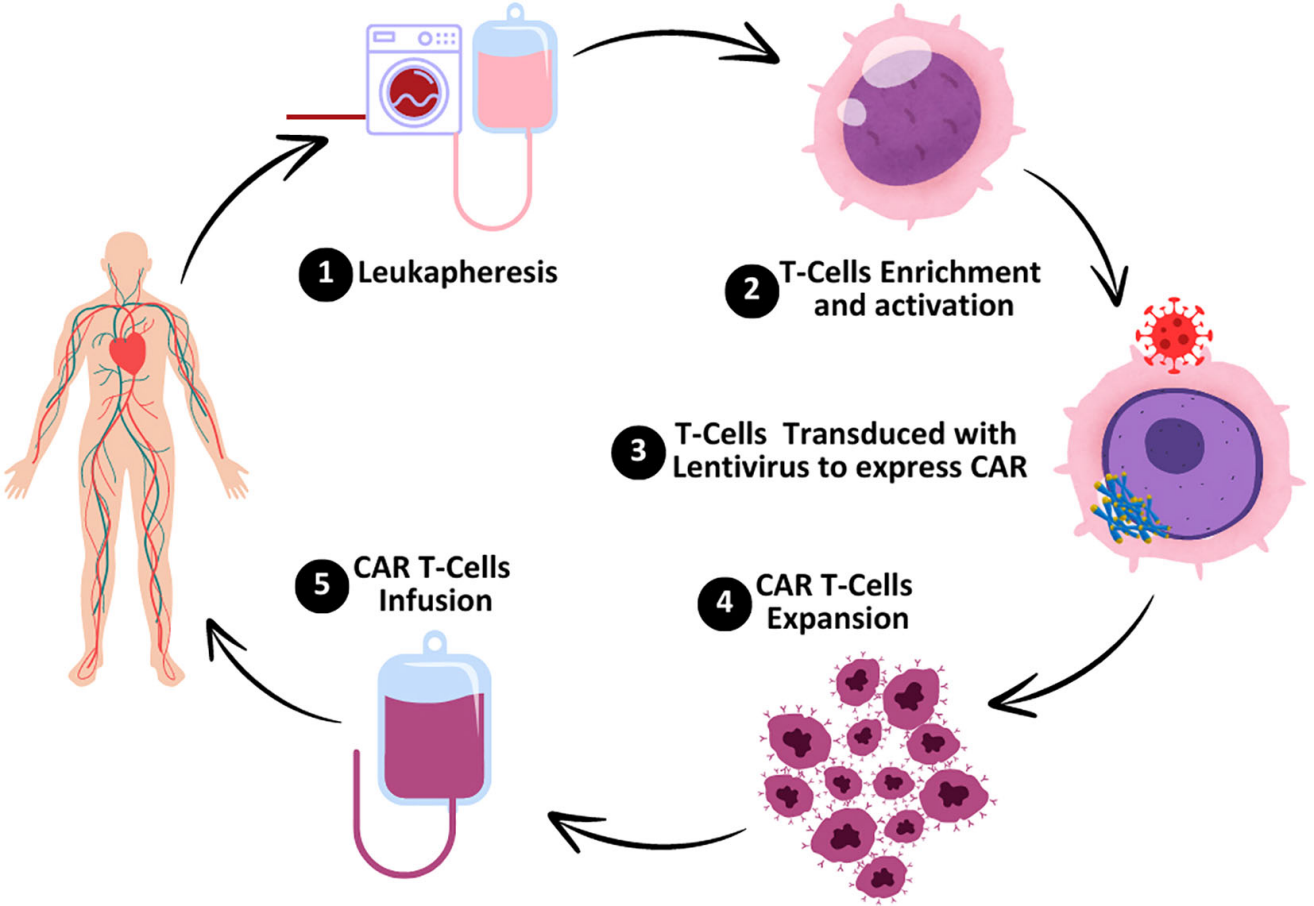
Preparation of CAR T cells. The process starts with leukapheresis, the process by which the peripheral blood is taken from the patient and T cells are isolated. T-cell enrichment and activation take place in the laboratory which enables the selection of T cells and stimulation. T cells are transduced with a lentiviral vector containing the gene of the chimeric antigen receptor (CAR) so that the T cell can identify and bind to specific cancer cells. In CAR T cells, there is a process of expansion where the cells divide and multiply in order to reach the necessary amount for administration. Finally, the genetically engineered CAR T cells are reintroduced into the patient’s body to locate and kill cancer cells.

A novel approach called chimeric antigen receptor (CAR) T-cell immunotherapy amplifies T cells to combat cancerous cells. This potent immunotherapy targets cancer cells through accurate tracking with minimum risk to the healthy cells of the human body (1). In this approach, the chimeric antigen receptor (CAR) gene is fused into the genomic structure of the host cell by a viral vector, such as a retrovirus or lentivirus (**Figure 1**). This fusion of the CAR gene develops sustained transgenic expression. Chimeric antigen receptors have the potential to detect, stimulate, and activate various receptor chains through the replication of genuine T cell receptors of a complex nature. CAR T cells can persist in the host circulatory system for a long duration and act as long-lasting memory cells. When cancer cells regenerate, they detect and eradicate them from the circulatory system (2).

**Figure 2:**
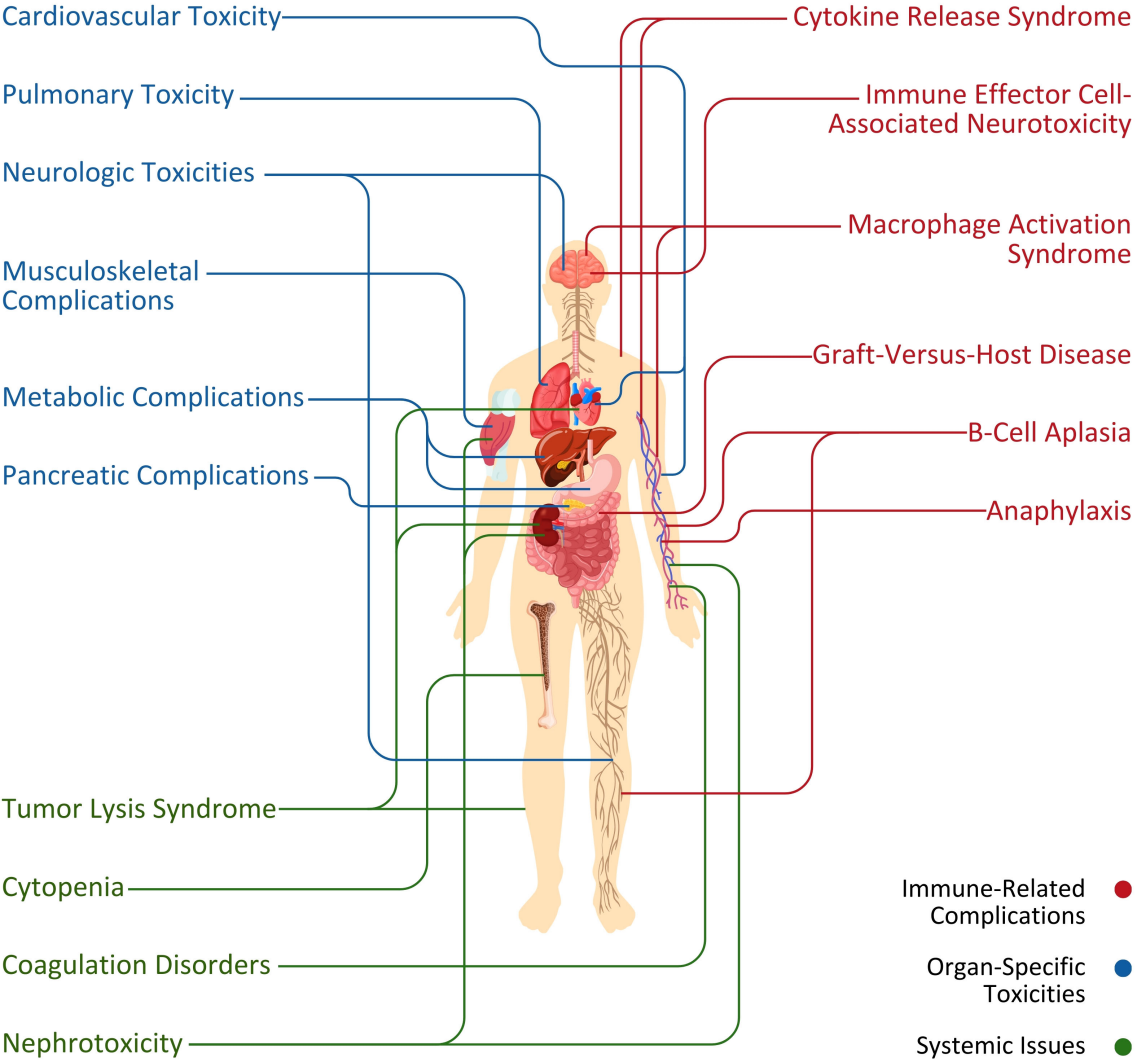
Association between primary systems and adverse events (immune-related complications, organ-specific toxicities, and systemic issues) initiated by CAR T-cell therapy.

Novel CAR T-cell therapy is widely considered a successful treatment against various blood and solid tumor malignancies. Despite that, its universal adoption is challenging due to adverse events (**Figure 2**) limiting the impressive early responses in clinical trials (3). In terms of the toxic effects (**Figure 2**) of this therapy, CAR T cells have particular toxicity due to their innate biological makeup (4). This study highlights the critical problems and toxicities resulting from CAR T-cell therapy and their revealed mechanisms are discussed to advance this approach or innovations in the domain of cancer treatment.

### 1.1 Cytokine release syndrome

**Figure 3:**
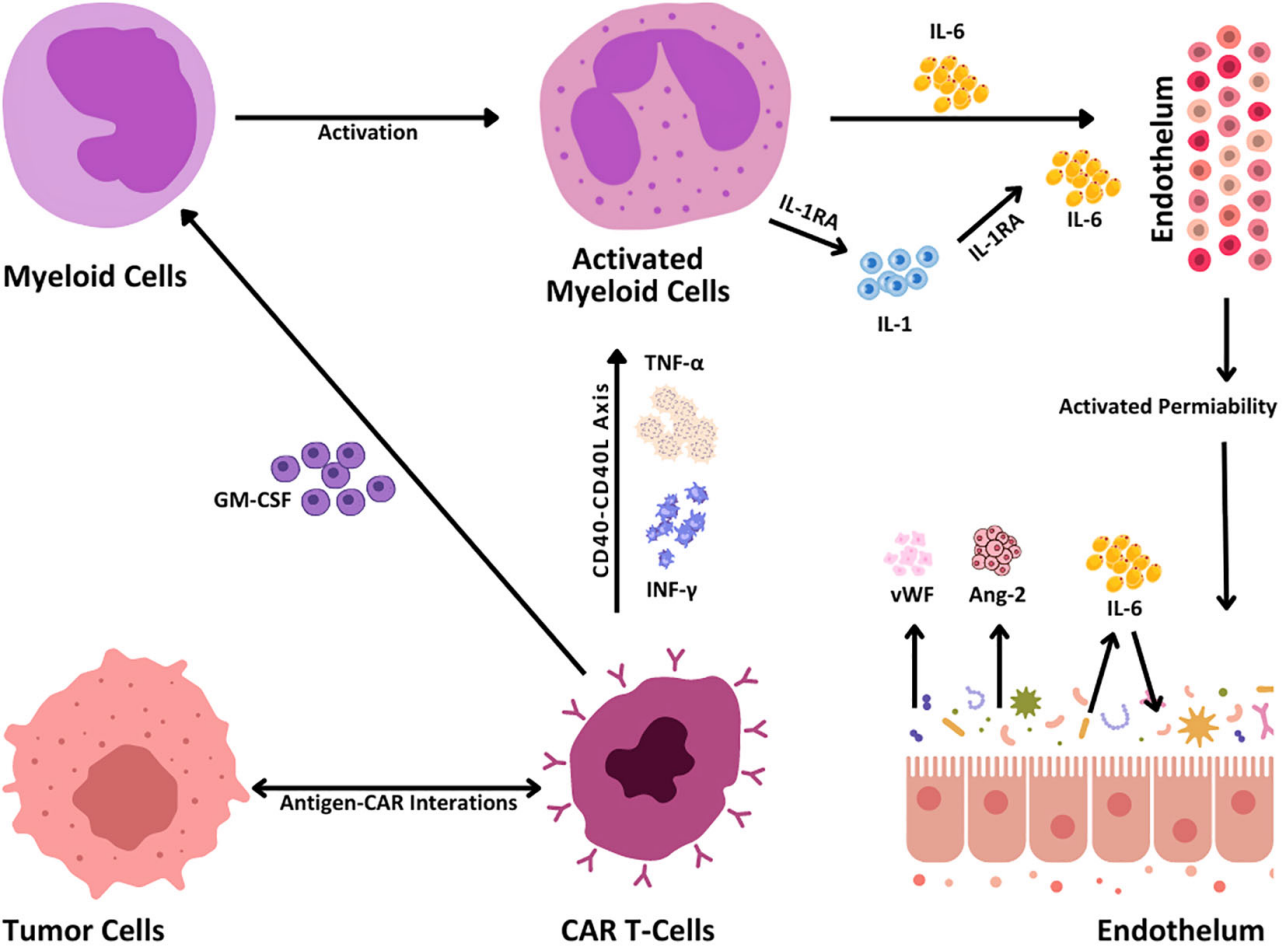
CRS mechanisms. When engaging the tumor cells, CAR T cells become activated via the interaction between antigens and CARs that leads to the production of inflammatory cytokines such as TNF-α and interferon-gamma (IFN-γ). This cytokine release in turn activates the CD40-CD40L signaling pathway, reacting more prospectively to immune activation. When secreting granulocyte-macrophage colony-stimulating factor (GM-CSF), CAR T cells activate myeloid cells, and subsequently IL-1, IL-6, and interleukin-1 receptor antagonist (IL-1RA) are hence secreted. These cytokines act upon the endothelium and result in enhanced permeability of blood vessels. Furthermore, the release of Von Willebrand factor (vWF), angiotensin 2 (Ang-2), and IL-6 promotes endothelial activation responsible for tumor growth and immune response.

The outstanding effectiveness of CAR T cells, however, has been associated with considerable potentially fatal toxicities, the most frequent of which is cytokine release syndrome (CRS) (5). CRS (**Table 1**) is caused by generalized immunological activation and is associated with significant increases in inflammatory cytokines such as granulocyte-macrophage colony-stimulating factor (GM-CSF), interferon-gamma (IFN-γ), tumor necrosis factor-alpha (TNF-α), and interleukin-6 (IL-6) (**Figure 3**). CRS is quite common, with a rate ranging from 60% to 93%, but grades 3 and 4 have rates as low as 13% to 14% (9). These increase the immune response via a cytokine chain that includes other immune cells. The subsequent cytokine storm causes elevated vascular permeability, activation of endothelial cells, and multiorgan dysfunction, which appears as hypotension, edema, neurotoxicity, and organ dysfunction (**Figure 3**). To manage CRS, medications such as tocilizumab target important cytokines, primarily IL-6, to diminish the inflammatory process and improve symptoms (6).

**Table 1:** Cytokine release syndrome.

**Pathogenesis**	T cells are activated when they recognize a tumor antigen
**Timing**	Symptoms might not appear until few days and even weeks following therapy, determined by the rate at which T cells activate (6, 7)
**CRS Grading**	Grade 1: Fever ≥ 38°C; Nausea; Flu-like symptoms Grade 2: Fever ≥ 38°C; Hypoxia requiring low-flow nasal cannula; Hypotension not requiring vasopressors Grade 3: Fever ≥ 38°C; Hypoxia requiring high flow or face mask; Hypotension requiring one vasopressor with or without vasopressin Grade 4: Fever ≥ 38°C; Hypoxia requiring positive airway pressure; Hypotension requiring multiple vasopressors (8)
**Mediator**	IL-6 is a crucial mediator
**Management**	Inhibiting the IL-6 pathway or using corticosteroids can alleviate symptoms

According to clinical trials, CRS initially causes fever in the patients following CAR T-cell treatment, and the fever duration is CRS grade-dependent (**Table 1**). Following the CAR T-cell therapy, patients with grade three or four CRS have a fever within 25 hours, while patients with a CRS grade lower than three experience a fever after 12 days (10, 11). Furthermore, the patients exhibit insufficient oxygen levels, low blood pressure, elevated heart rate, and various neurological abnormalities including language difficulties, impaired handwriting, and reduced focus abilities (12, 13). The levels of IFN-γ, interleukin-2 receptor subunit alpha (IL2Ra), and soluble IL-6 serum indicators were found to determine the severity of CRS through a significant rise in severe CRS cases compared to those without the same severity (14). Severe CRS exhibits symptoms such as leakage of blood vessels, heart issues, impaired kidney function, accumulation of fluid in the lungs (pulmonary edema), abnormalities in blood clotting (coagulopathy), and hepatic failure (15). Park et al. (16) found severe CRS in 41% of patients with high disease stress, while only 5% of individuals with low disease stress exhibited CRS.

Early clinical studies using cluster of differentiation 19 (CD19) CAR T cells revealed considerably significant toxicities compared to the symptoms experienced in conjunction with further cellular therapies, demonstrating substantially widespread stimulation of the immune system (**Table 2**). The severity of CRS can vary significantly, with some patients experiencing mild symptoms while others develop life-threatening conditions (7).

**Table 2:** Fatality percentage for major issues induced by CAR T-cell therapy.

Major issues	Prevalence	Observed patients	Fatality	References
**CRS**	Oklahoma City, USA	1	After 12h	Afzal et al. (9)
	Pittsburgh, Pennsylvania	1	After 2 months	Marker et al. (6)
	USA	1	After 9 days	Pemmaraju et al. (17)
	Paris, France	48	22.92%	Belin et al. (18)
	Hannover, Germany	15	No	Möhn et al. (19)
	Victoria, Australia	53	No	Sales et al. (20)
	France	238	4.8%	Le Cacheux et al. (21)
	Washington, Pennsylvania	43	7%	Gazeau et al. (22)
	Rennes, France	190	No	Mauget et al. (23)
	California, USA	26	3.84%	Nie et al. (24)
	California, USA	359	39%	Locke et al. (7)
	USA	148	3%	Jacobson et al. (25)
	USA	269	0.37%	Abramson et al. (26)
	USA	57	5.26%	Shah et al. (27)
**ICANS**	Paris, France	48	22.92%	Belin et al. (18)
	Hannover, Germany	15	No	Möhn et al. (19)
	Victoria, Australia	53	No	Sales et al. (20)
	France	238	4.8%	Le Cacheux et al. (21)
	Washington, Pennsylvania	43	7%	Gazeau et al. (22)
	California, USA	1	No	Nie et al. (24)
	New York	3	No	Santomasso et al. (28)
	Rennes, France	190	No	Mauget et al. (23)
	Maryland, USA	79	No	Shalabi et al. (29)
	Xuzhou, China	60	No	Wang et al. (30)
**TLS**	Chicago, USA	1,595	12%	Obeidat et al. (31)
	Chicago, USA	8,779	32.3%	Moturi et al. (32)
	USA	9,034	32%	Gangani et al. (33)
	Mexico, USA	138	30.4%	Rios-Olais et al. (34)
	Arkansas, USA	1,808	19.7%	Roy et al. (35)
	Inglewood, Canada	930	14%	Adla Jala et al. (36)
	Shaanxi, China	480	17%	Feng et al. (37)
	Phoenix, Arizona	246	65.78%	Kelkar and Wang (38)
	Beijing, China	164	43.29%	Wang et al. (39)
	Istanbul, Turkey	107	12.38%	Bozkurt et al. (40)
	Karachi, Pakistan	400	36%	Ahmed et al. (41)
	USA	141	2.1%	Cairo et al. (42)
**MAS**	Minnesota, USA	5	2 died	Monteagudo et al. (43)
	Geneva, Switzerland	1,080	13.7%	Amikishiyev et al. (44)
**B-Cell Aplasia**	Spain	23	3 died	Molinos-Quintana et al. (45)
	Stanford, Canada	41	2.44%	Baird et al. (46)
	London, United Kingdom	151	4%	Gabelli et al. (47)

New developments (48) in managing CRS in the past few years include knowledge about the molecular pathways and the evolution of the approach to their management, which is significant in the context of CAR T-cell therapy. This is mainly orchestrated through the activation of cytokines such as IL-6 and IL-1 from the immune cells, especially the macrophages and the monocytes. These cytokines cause the dangerous inflammatory reactions that define CRS: developed fever, hypotension, and organ damage. Existing approaches (4, 49) are focused on reducing the off-target effects without any impact on the effectiveness of CAR T-cell therapy. Calcineurin inhibitors are still strong cornerstones in the treatment of CRS and tocilizumab, an IL-6 receptor antagonist, still plays a big role in its management. Inflammation decreases and patients’ conditions improve due to the use of this treatment, though it is more useful in patients with mild to moderate CRS. However, IL-1 is likely to have a critical role in both CRS and its neurotoxic assets, which is referred to as cytokine release syndrome-associated encephalopathy syndrome (CRES). However, another anti-interleukin-6 receptor (IL-6R) molecule, anakinra, an IL-1 receptor antagonist, appears useful in CRS prevention and other neurotoxicities that may not be treated by tocilizumab. Moreover, experiments with animal models identified that the suppression of nitric oxide synthase (NOS), which is enhanced during severe CRS, may eliminate life-threatening hypotonia and other toxic side effects. This has paved the way for the administration of nitric oxide synthase inhibitors as supplementary treatment, in order to enhance the patient prognosis in complicated cases. There are also changes in prophylactic treatment. Steroids and IL-6 blocking for prevention were thought to be counterproductive because they would suppress the anti-tumor effects of CAR T cells, however, this has recently been shown not to be the case when using anti-IL-1 or anti-IL-6 treatment. This has influenced the changes in clinical practice whereby tocilizumab or anakinra are administered early in an attempt to decrease CRS-associated morbidity and mortality with no negative impact on cancer treatment.

### 1.2 Immune effector cell-associated neurotoxicity syndrome

Another common side effect of CAR T-cell therapy is immune effector cell-associated neurotoxicity syndrome (ICANS). It was once named CRES, chimeric antigen receptor T-cell-related encephalopathy, or neurotoxicity alone (12). When cluster of differentiation 28 (CD28) is used as a costimulatory domain in CAR constructs, high-grade ICANS is often also present, affecting up to 45% of treated patients (50). CAR T cells striking cluster of differentiation 22 (CD22), B cell maturation antigen (BCMA), and other hematological antigens have been shown to cause neurotoxicity, in addition to CD19. The same neurotoxic outcomes have been documented concerning alternative immune effector cell (IEC) therapies, including blinatumomab (51). ICANS often causes impairment of concentration and disorientation. Expressive aphasia and alterations in handwriting are regarded as very specific and initial manifestations of ICANS. It usually leads to lowered consciousness and disorientation, leading to coma, convulsions, motor/muscular weakness, and cerebral edema. CD19-associated CAR T-cell therapy with 4-1BB or CD28 constructs has resulted in severe neurotoxicity owing to cerebral edema (3% incidence). All fatal cerebral edema cases were linked to CRS, and severe CRS was linked to severe ICANS (3).

ICANS can manifest as a range of neurological symptoms, including confusion, delirium, seizures, and cerebral edema. The onset and severity of ICANS can vary (**Table 2**), with some patients experiencing mild cognitive changes while others suffer from severe and potentially fatal cerebral edema (18). Histopathological findings in cases of fatal ICANS often reveal neuronal death, neuronal and perivascular edema, and intraparenchymal hemorrhagic extravasations (21). According to Le Cacheux et al. (21), ICANS can be managed through supportive care and corticosteroids. Regular neurological assessments and advanced neuroimaging are important for the early detection and management of ICANS. In some cases, antiepileptic medications can also be utilized to manage seizures and other severe neurological symptoms that appear following CAR T-cell therapy.

Current investigations regarding ICANS resulting from CAR T-cell therapy exhibited a lower incidence, however, clinical studies reported intermediate to catastrophic ICANS in 30% to 60% of individuals (20, 25). In the study by Belin et al. (18), the mortality rate resulting from ICANS was modest. Nevertheless, the early detection of moderate to severe illness is imperative since patients frequently require extended hospitalization. Despite the variability, prompt detection is important to extend hospitalization due to the severity of the illness associated with moderate to severe ICANS. The low mortality rate (0% to 1.4%) of ICANS indicates the importance of appropriate and prompt treatment to avoid unfortunate results (20).

Clinicians completely understand the management of ICANS, including patients who have undergone chimeric antigen receptor CAR T-cell therapy in recent years. However, the management of ICANS is sometimes done in conjunction with the management of CRS (52) because they affect patients simultaneously. The first line of treatment for ICANS is the use of corticosteroids, specifically dexamethasone, once neurological signs are observed. For the scenarios that do not show an improvement with the first cycle of steroids, the administration of methylprednisolone in an increased dosage may be recommended. Recent studies (53) have discussed the use of an IL-1 blockade by anakinra in the treatment of steroid-unresponsive ICANS. Anakinra, an IL-1 receptor antagonist that treats neurotoxicity, has been effective in treating those patients who do not benefit from conventional management. This therapeutic approach is especially useful in severe or persistent ICANS where inflammation processes should be stopped as soon as possible to avoid devastating outcomes for the patient. Other adjunctive therapies such as antiepileptic drugs (AEDs) and constant supervision, especially in the Intensive Care Unit, are important in the management of severe cases. However, investigations are being conducted into measures to prevent the development of ICANS. These include pretreatment with agents such as anakinra or newly developed treatments that selectively address the inflammatory processes more efficiently.

### 1.3 Tumor lysis syndrome

Tumor lysis syndrome (TLS) is induced by anticancer therapies for tumor cell lysis that develop metabolic content constellations that are dispersed into the bloodstream. Such constellated metabolic content can result in medical conditions such as hyperkalemia, hyperphosphatemia, and hyperuricemia (54, 55). Since TLS is induced by denatured cellular components, its development is directly proportional to the tumor cell proliferation speed or size of the tumor burden (56). Moreover, high levels of lactate dehydrogenase (LDH) with fever are also reported in the development of TLS in some patients (57).

TLS is a CAR T-cell therapy-induced complication that can lead to renal failure, arrhythmias, and fatality (**Table 2**) due to the destruction of cancerous cells by direct CAR T-cell therapy or lymphodepleting chemotherapy (3, 58). Patients treated with CAR T-cell therapy without lymphodepletion chemotherapy have also been reported to have TLS (59). Hence, in cases of high tumor burden according to TLS, prophylaxis as per standard medical guidelines and the use of hypouricemic agents (febuxostat, rasburicase, and allopurinol) should be employed before administering CAR T-cell therapy or initiating lymphodepleting therapy (3).

New developments in TLS treatment emphasize the prediction of TLS and prompt treatment to avoid complications. TLS is considered a potentially fatal condition and is most commonly associated with hematological malignancies such as acute lymphoblastic leukemia (ALL) and high-grade lymphoma. For high-risk patients, other preventive treatments (60) such as adequate hydration and administration of drugs that reduce serum uric acid levels, are critical. Xanthine oxidase inhibitors are described and remain an effective first-line strategy to protect the patient against hyperuricemia by blocking the synthesis of uric acid. Another drug in the category of xanthine oxidase inhibitors is febuxostat but it is rarely used because of its high cost and its side effects that are perilous in patients with cardiovascular conditions. A recombinant urate oxidase, rasburicase, assumes a central role in the management of developed TLS by reducing uric acid to the more soluble allantoin. This agent lowers the uric acid level much faster than any other agent and is therefore commonly recommended for patients with high-risk disease or preexisting hyperuricemia. Published literature (61) shows newer approaches to preventive and curative measures depending on the features of risk such as tumor load, impaired kidney function, or malignancy type. Additionally, the monitoring of urine output and managing electrolyte disturbances such as hyperkalemia is very important in managing TLS. Agents such as phosphate binders and sodium zirconium cyclosilicate have been used to address problems such as hyperphosphatemia and hyperkalemia, respectively. Despite improvements in pharmacological options for modification of outcome, the concept of managing TLS has remained primarily preventive rather than curative through monitoring and early intervention.

### 1.4 Macrophage activation syndrome

**Figure 4:**
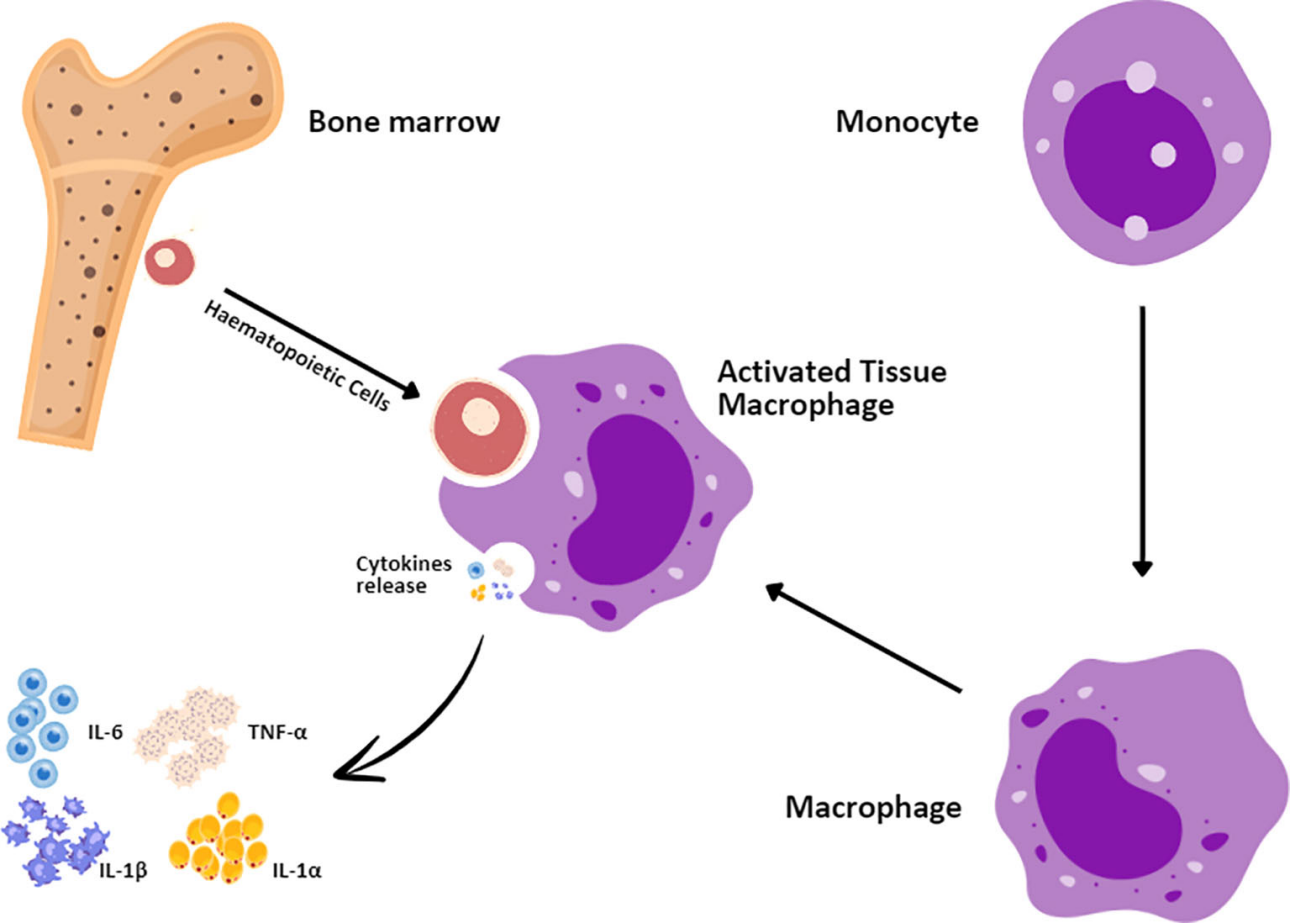
Macrophage activation syndrome mechanism. Hematopoietic cells in the bone marrow interact with monocytes, which enter the bloodstream before settling in tissues where they develop into macrophages. The first responders to injury are the tissue macrophages that upon activation secrete a number of proinflammatory cytokines, such as IL-1α, interleukin-1 beta (IL-1β), IL-6, and TNF-α.

Autoimmunity-associated hemophagocytic lymphohistiocytosis (HLH) is referred to as macrophage activation syndrome (MAS) (3, 62). MAS is a menacing problem that arises from the excessive proliferation and hyperactivation of T lymphocytes and macrophages (**Figure 4**) (55). Patients receiving CAR T-cell therapy also experience the symptoms of HLH/MAS (50, 63) due to its parallel criteria with grade 3 CRS. HLH/MAS associated with CAR T-cell therapy has been managed with CRS treatment including tocilizumab and corticosteroids (64, 65). According to Maude et al. (66), it was ambiguous whether MAS/HLH is a specific toxic entity or appears as a result of CRS hyperinflammation. HLH/MAS diagnostic criteria associated with CAR T-cell therapy were defined by the elimination of HLH/MAS from the terms of CRS following the ASTCT grading guidelines (3, 12). The proposed treatment, including etoposide and methotrexate or intrathecal cytarabine for refractory HLH/MAS, is still controversial. Administration of anti-IL-1 with anakinra has also been proposed for HLH/MAS treatment but has not been approved for the management or treatment of HLH/MAS (3, 14, 66).

### 1.5 B-cell aplasia

B-cell aplasia and hypogammaglobulinemia are anticipated issues after CD19-directed CAR T-cell therapy because of the off-tumor-on-target effect of CD19-associated CAR T cells on natural B cells. CAR T-cells with 4-1BB as a secondary pathway lead to chronic B-cell aplasia up to 5 years after treatment by CAR T-cells for ALL (67, 68). Thus, the persistence of CAR T-cells can be predicted through B cell levels as pharmacodynamic biomarkers. Remission duration has a potential role in B-cell recovery for ALL (68–70). However, during perpetual remission after CAR T-cell therapy, recovery of B cells in lymphoma can manifest (71, 72).

Severe infections are associated with hypogammaglobulinemia developed from B-cell aplasia (67). During B-cell aplasia, empiric immunoglobulin (Ig) replacement in pediatric patients is executed on a standard basis (68, 73). Different approaches to Ig replacement and CD19-negative memory plasma cells (that secrete antibodies) that increase basic humoral immunities have been described to treat B-cell aplasia and hypogammaglobulinemia in adults due to CAR T-cell therapy (74, 75).

### 1.6 Anaphylaxis

The non-human elements of most CAR constructs pose a risk to clinical efficacy and could lead to allergic responses. CAR T-cell infusion followed by anaphylaxis has been published but allergic responses are rare after frequent CAR T-cell therapies (76). However, CAR T-cell therapy with tisacel and axicel has been reported to lead to the production of preexisting anti-murine antibodies against CD19-directed CARs in patients (69). Although CD-19 antibodies associated with rising titers against CARs have been revealed, the expansion, efficacy, and persistence post-infusion of CAR T-cells are genuine. Fully human generated CARs to minimize immunogenicity have still not been reported (72, 77).

New developments in anaphylaxis and immunogenicity management (78, 79) in the current population underline the need for early detection, individual therapeutic approaches, and novel methods of monitoring. The management of anaphylaxis (80) has progressively shifted more toward the search for biomarkers, primarily serum tryptase, that facilitate speedy diagnosis and risk assessment. We, therefore, recommend the use of additional tryptase levels immediately after the onset of anaphylactic reactions to inform emergency management. Immunogenicity, especially in biological products and monoclonal antibodies, remains an issue thanks to the development of antidrug antibodies (ADAs). These ADAs can decrease the therapeutic effectiveness of the active substance and cause hypersensitivity reactions up to anaphylaxis. Some of the new approaches that are seen at the developmental stage relate to computational and *in vitro* modeling of immunogenic risk early in drug development. Immunogenicity is traditionally a highly litigious issue and derisking immunogenicity through methods such as T-cell epitope mapping and high-end *in silico* algorithms is now getting deployed more and more for late preclinical and early clinical validation (81). Furthermore, factors that can be related to the individual patient, for instance, the patient’s heredity and previous treatment with related biologics, are included in the risk evaluation tools used to identify possible immunogenic reactions. Some strategies aimed at determining immunogenicity include using fully humanized or engineered antibodies so as to avoid immune identification. Further, in clinical management, it is common to prescribe antihistamines and corticosteroids before the procedure in patients who are sensitive to latex. It is crucial to enhance patient prognosis in therapeutic endeavors and to continue research on the molecular base of ADA formation and hypersensitivity reactions.

### 1.7 Graft-versus-host disease

CAR T-cell therapy has the potential to bind T cells to protein, carbohydrate structures, and glycolipids and contributes to the persistence and expansion of T-cells. It can be active in both CD8+ and CD4+ cells and there is a low chance of graft-versus-host disease (GVHD) and autoimmunity (82). GVHD, with adverse effects on the vital organs of recipients of CAR T-cell therapy, is an immune response and can be mitigated by immunosuppressive medication (83). GVHD has not been considered a threatening issue in patients posttransplant since this therapy was not accepted for the treatment of ALL in the early days (55). However, only one report of chronic GVHD with drastic skin GVHD 3 months after the CAR T-cell therapy has been published. Subsequently, corticosteroids were administered for the management of the incidence of GVHD (84).

### 1.8 Acute myelofibrosis

The investigations of Lai et al. (85) revealed acute myelofibrosis (AMF) as a serious threat that appeared by CD-19-associated CAR T-cell treatment, beyond the neurotoxicity and CRS. After receiving CD19 CAR T-cell therapy, 1 out of 17 patients diagnosed with B-cell acute lymphoblastic leukemia (B-ALL) exhibited AMF following grade IV CRS. Despite achieving complete remission (CR) from B-ALL, a 36-year-old male patient with grade IV CRS and Philadelphia chromosome-negative B-ALL eventually had a fatal relapse through worsening of bone marrow fibrosis. It was also concluded by primary myelofibrosis (PMF) mutations such as JAK2, MPL, and CALR that pre-existing genetic predisposition was not the reason for AMF. A CRS-induced cytokine milieu with IL-6 among other profibrogenic and inflammatory cytokines was the cause of AMF development (85). This was in line with the cytokine expression and inflammatory response involved in myelofibrosis.

The treatment of AMF has been recently improved (86) with an emphasis on combination therapy and new drugs. Ruxolitinib is and still remains an essential part of the management of this disease as it has been proven to be very effective in managing symptoms such as splenomegaly. Recent studies (87), however, show that an increasing number of studies have pointed towards the superiority of combining JAK inhibitors with non-JAK agents in the spleen with symptom control and reasonable tolerability. Further, the newer agents that have emerged, fedratinib, pacritinib, and momelotinib, have brought more options to the table particularly in managing patients with anemia and thrombocytopenia. Of these, momelotinib has emerged as potentially clinically effective in treating anemic AMF patients and improves their symptoms and transfusion dependence compared with conventional therapies. Currently, clinical studies are still being conducted to further understand other combinations and doses that are safe and effective for the patients with managed side effects.

### 1.9 Cytopenia

Cytopenia, including neutropenia, anemia, and thrombocytopenia, is a common chronic side effect of CAR T-cell treatment that adversely impacts the immune systems of patients (88). Alarmingly, prolonged cytopenia for 3 months or above has been observed following CAR T-cell infusion, and 15% of patients were diagnosed with B-cell lymphoma (89). Patients are also affected by cytopenia in continuous absolution with no confirmation of myelodysplastic syndrome. However, the actual mechanisms behind these prolonged cytopenias have not yet been reported (90).

In some reports of CAR T-cell therapy, cytopenia was common after 4 to 39 months of CAR-T cell infusions but they characterized cytopenia as myelodysplastic syndrome (MDS) (91, 92). Such confusion raises the importance of ruling out the mechanisms of MDS or CAR-T therapy as the sources of cytopenia (93). Later, it was confirmed by Strati et al. (91) through statistical MDS diagnosis that cytopenia at day 30 after CAR T-cell infusion was not associated with myelodysplastic syndrome. Meanwhile, it was reported by Jain et al. (94) that inflammation factors were significantly associated with hematopoietic recovery at 1 month and this imbalanced the observations about cytopenia.

After CAR T-cell therapies, grade 3 cytopenia is frequently reported. Febrile neutropenia of grade 3 was recorded in 17% and 31% of the patients examined within the JULIET trials and ZUMA-114, respectively (95, 96). Almost 30% of patients following CD19-specific CAR T-cell therapy with tisacel or axical exhibited prolonged severe cytopenia in a biphasic pattern beyond 30 days after administration (71, 97). Nevertheless, the etiology of late cytopenia is not fully described with the lymphodepleting chemotherapy being considered to be associated with early cytopenia (97, 98). Whereas, in severe CRS, prior hematopoietic cell transplantation (HCT), and frequent prior chemotherapies are attributed as the cause of late cytopenia (11, 96, 97). Patients with limited hematopoietic capacity who underwent a prior HCT showed a disturbance in their chemokine milieu following CAR T-cell therapy, and CAR-specific immunobiology has been described as a fundamental issue (11, 97). Platelet and erythrocyte replacement have been used in the treatment of thrombocytopenia and anemia. Similarly, patients with prolonged neutropenia should be treated with granulocyte colony-stimulating factor (G-CSF) (8, 99). However, according to previous studies, it might enhance the severity or incidence of CRS due to a lack of immune cell activation. However, CAR T-cell therapy can be useful as initial care in patients (99, 100). Anecdotally, the transfusion of allogeneic or autologous stem cells resolved the persistence of cytopenia after CAR T-cell therapy (96, 101).

### 1.10 Coagulation disorders

**Figure 5:**
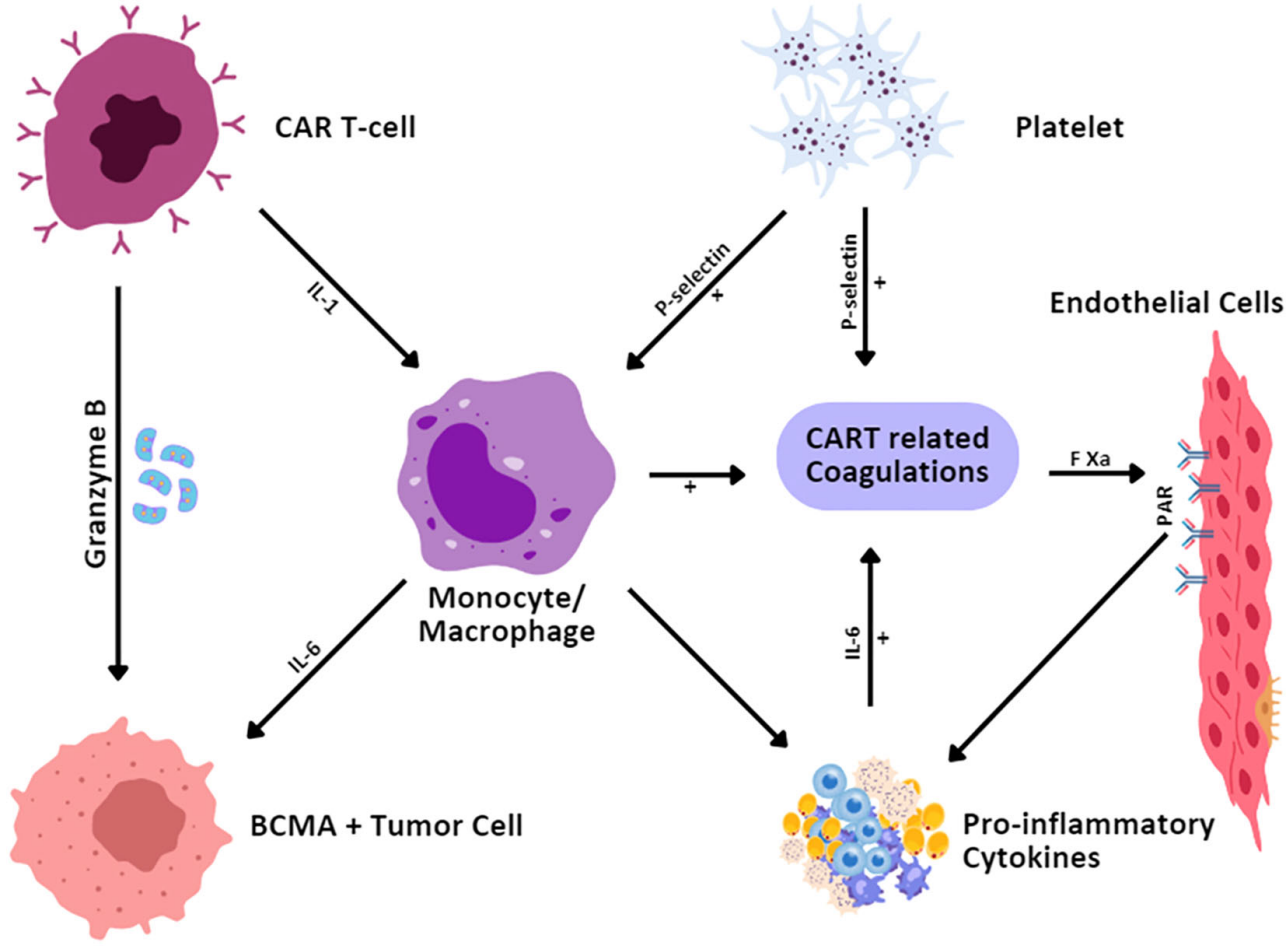
Mechanisms for CAR T-cell-therapy-related coagulation disorder. CAR T cells, upon identifying and binding to the BCMA-expressing tumor cells, secrete granzyme B to cause the death of the tumor cells. This interaction also initiates cytokine secretion by monocytes/macrophages including IL-1 and IL-6. IL-6 stimulates increased cytokine activity and inflammation and also activates endothelium and platelets. The expression of P-selectin on platelets and endothelial cells contributes to coagulation. Furthermore, activated endothelial cells through Factor Xa (FXa) and protease-activated receptors (PAR) are involved in the coagulation of CAR T cells and strengthen the inflammatory process.

During the administration of CAR T-cell therapy, coagulation disorders, particularly hematological malignancies, have been observed in 51%-56.6% of patients within 6 to 20 days after the infusion (30, 102). Increased fibrinogen degradation products, decreased fibrinogen, increased D-dimer, thrombocytopenia, and prolonged prothrombin time are initiated by CAR T-cell therapy-associated coagulation disorders. Since the appearance of disseminated intravascular coagulation (DIC) is associated with severe coagulation disorders, only a few cases have been published regarding CAR T-cell therapy-associated DIC incidence (**Figure 5**). Various reports indicated an incidence of DIC of approximately 7% to 28.3% in patients following CAR T-cell infusion (30). In addition, the grade of CRS positively impacts the coagulation disorder’s severity (30, 103). Patients with severe CRS have also been reported to have a higher incidence of DIC and coagulation disorders (102).

Conventional approaches for the treatment of patients with coagulation disorders are generally used for standard care and most of the patients are observed to recover without intervention (30). The development of DIC and the levels of multiple cytokines are decreased or eventually inhibited by the management of CRS (103, 104). Effective and timely treatment and interventions are essential once DIC symptoms appear in patients. New developments (105) in care and control strategies for coagulation syndrome are based on the targeted intervention model. The early use of fibrinogen concentrates and antifibrinolytic agents has also demonstrated benefits in the treating of trauma (TR)-induced coagulopathy. Current attitudes toward the use of anticoagulants, especially in critically ill patients (106), are more focused on the choice between thrombotic and bleeding events; more novel antithrombotic agents and reversal approaches are needed. Coagulation disorders can be avoided with the help of normothermic management, correction of acidosis in the body, and normalization of calcium levels for the thickening of fibrin strands that support coagulation.

### 1.11 Other cascade complications

The expression of tumor antigens, which is targeted in CAR T-cell treatment, is essential for effective targeted therapy. Such antigens, known as tumor-specific antigens (TSAs), should be visible to CARs on tumors. CAR T-cell therapy mainly targets tumor-associated antigens (TAAs) since TSAs are few during treatment (103). Tumor cells and normal cells indicating the target antigens are identified and killed by infused CAR T cells. This mechanism to kill the malignant cells appearing on normal cells is a known tissue-on-target effect that sometimes leads to serious complications and mortality. CAR T cells targeting ERBB2 (HER-2/neu) were developed by Morgan et al. (107) for the treatment of cancer patients with ERBB2 overexpression. Their team infused this treatment in one patient with liver and lung-propagated colon cancer and recorded respiratory distress within 15 minutes of infusion and pulmonary infiltration after chest x-ray analysis. The patient died within 5 days despite aggressive treatment. Later, according to the researchers’ hypothesis, after infusion, CAR-T cells penetrated the lungs and targeted lung epithelial cells exhibiting low ERBB2 expression. This process resulted in CRS by inducing the release of a large number of cytokines. To reduce off-target effects, the utilization of TSAs to induce the respective CAR T cells is a potent approach but it is expensive and challenging to find new TSAs (103). Thus, studies are needed for the optimization of CAR T cells through the structure of the CARs by using novel techniques, particularly inhibitory CARs (iCARs) and synNotch receptors (103, 108).

#### 1.11.1 Cardiovascular toxicity

Initially, cardiovascular toxicity following CAR T-cell therapy was observed in children suffering from ALL. In more than 5% of patients, pulmonary edema, hypotension, and fluid overload were observed, which are grade 3 cardiovascular toxicities, during the ELIANA trials (109). Additionally, retrospective analyses revealed cardiomyopathy causing left ventricular systolic disorder. In some cases, CAR T cells reversed such toxicities within weeks to months in most children (110). Two studies showed that CAR-T cell infusion resulted in acute cardiac tamponade and pericardial effusion through CRS (111, 112). These two cases were managed by eliminating CRS using dexamethasone and tocilizumab and one mortality was noted (112). Another clinical study of B-cell lymphoblastic lymphoma detected cardiac tamponade 5 months after developing grade 2 CRS (113). The studies of Tao et al. (114) reported acute cardiovascular events in a 76-year-old patient with diffuse large B-cell lymphoma (DLBCL) during CAR T-cell infusion. Following autologous CAR-T therapy, a patient receiving CD-19 CAR T-cell therapy exhibited CRS-associated coronary vasospasm. Moreover, a few studies revealed that 13.3% of individuals with cardiovascular toxicity were associated with CAR T-cell therapy (115, 116).

Approved CAR T-cell constructs have been reported in two retrospective analyses. After CAR T-cell therapy, it was observed in 17% of patients that cardiovascular events usually occurred for 1 month (117). In parallel, among 60 consecutive adult patients of LBCL treated either with tisagenlecleucel or axicabtagene ciloleucel after CAR T-cell infusion, 32 patients exhibited 48 cardiovascular adverse events within 1 year (118). Fluid retention, atrial fibrillation, and hypotension have also been observed with cardiovascular toxicities in a pediatric population. Notably, patients with CRS normally have cardiovascular events, which confirms the CRS association with cardiovascular damage (109, 117).

#### 1.11.2 Pulmonary toxicity

Pulmonary complications are common in immunotherapies, such as checkpoint inhibitor therapies. In recipients of CAR T-cell therapy, pulmonary toxicities in most of the cases have been revealed to date. However, patients with severe CRS exhibit frequent pulmonary toxicities (118) with particular symptoms including pneumomediastinum, hypoxia, allergic rhinitis, pleural effusion, and pulmonary embolism (109, 118). However, in some patients who received a CAR T-cell infusion, no comprehensive analysis has been seen for lung toxicity, and there are no comprehensive long-term evaluation reports for the transfer capacity of lungs after CAR T-cell therapy.

Haas et al. (119) treated two cases with solid tumors with CAR-T therapy targeting mesothelin (MSLN) and observed severe pulmonary toxicities. Within 48 hours of the CAR T-cell infusion, symptoms consistent with CRS and hypoxemia were seen and grade 5 respiratory failure was revealed in one patient. CAR T-cell accumulation, acute lung injury, and extensive T-cell infiltration in the lungs were also observed in the autopsy findings. Further evaluation showed low levels of MSLN in the benign pulmonary epithelial cells of lungs in fibrotic or inflammatory conditions. This observation revealed that lung pneumocytes contribute to dose-limited toxicity rather than pleural cells (115).

#### 1.11.3 Neurological toxicities

CAR T-cell therapy leading to toxicities that jeopardize the nervous system has captured the attention of numerous researchers seeking to develop this immunotherapy as a safe treatment. Severe IL-6, IFN-γ, and TNF-α levels at the beginning of the CAR T-cell therapy can result in neurotoxicity development with grade 3 or above (55, 84). According to univariate logistic analysis, the concentration of IL-6 is also a cause of grade 3 or higher neurotoxicity development (84). However, many reports demonstrated anti-cytokine therapies consisting of tocilizumab to prevent toxic neurological effects but resulted in no correlation between the neurotoxicity occurrence and CRS severity (55). Migration of cerebrospinal fluid (CSF) in the respective patients was determined as the main cause of the correlation between the administration of CAR T-cell therapy and the development of neurotoxicity due to their migration (70). Tremors, encephalopathy, headaches, hallucinations, confusion, and seizures are some neurological complications that influence the efficacy of CAR T-cell treatment (55, 70, 120). In a study by Turtle et al. (84), the total escape of the neurological issue over time was significant except for one fatality report.

#### 1.11.4 Genotoxicity

CAR T-cells are manufactured by the transduction of lymphocytes and viral vectors are utilized for the transduction process. Such viral particles are the cause of insertional mutagenesis (IM). When retroviral and lentiviral vectors transduce hematopoietic stem cells, IM is seen, but to date, no genotoxicity has been reported in differentiated cells through gene transfer (3, 121). Similarly, patients treated with manipulated T cells did not indicate any transformational event associated with retroviruses (122). However, in a patient with ALL receiving CAR T-cell therapy, the transduction of a leukemic B cell resulted in developing leukemia, relapse, and prompt death (123). After CAR T-cell therapy, non-melanoma skin cancer, bladder cancer, and myelodysplastic syndrome are reported secondary malignancies, but in some cases, the development of these malignancies is considered to be associated with previous therapies (93, 95). Overall, a longer observation period of almost 15 years will be required for CAR T-cell therapy toxicity data collection and evaluation, and the authorization of commercially available constructs (3).

#### 1.11.5 Musculoskeletal complications

CAR T-cell therapy is associated with musculoskeletal complications such as myositis and is characterized by muscle inflammation. CAR T-cell infusion also elevates creatine phosphokinase (CPK) levels which cause both weakness and muscle pain. Following CAR T-cell therapy, myalgias associated with CRS is a common clinically adverse event that influences the muscular system (55, 120).

#### 1.11.6 Metabolic complications

After CAR T-cell therapy metabolic toxicities have been recorded as a frequent complication in 60 patients by Wudhikarn et al. (118). Hypophosphatemia, hypocalcemia, and hypokalemia were common electrolyte abnormalities observed after CAR T-cell Infusion. Hypoglycemia and hyperglycemia have also been reported frequently [Penack and Koenecke, (109)]. However, such complications are not persistent and can be managed.

#### 1.11.7 Pancreatic complications

Complications observed in the pancreas after CAR T-cell therapy should be considered. However, there are few published reports on such toxicities in the literature. Pancreatic complications have been described due to the development of pancreatitis in five patients after the administration of CAR T-cell therapy (120).

#### 1.11.8 Nephrotoxicity

Clinical syndromes causing acute kidney injury (AKI) are characterized by low renal excretions caused by sepsis and the aggregation of nitrogen-based metabolic products (55). This toxicity has rarely been reported due to CAR T-cell adverse events and has been found in stage 2 to 3 syndromes with renal electrolyte imbalances and other renal toxicities (59, 120).

### 1.12 How to avoid cascade complications

Avoiding cascade complications is important in cardiovascular and pulmonary conditions, especially in subjects with comorbid conditions where early evaluation and management are important. Screening with echo, electrocardiography (ECG), and pulmonary function tests (PFTs) is useful for identifying at-risk subjects in the population. Where the patient has had prior heart failure, reduced left ventricular ejection fraction (LVEF), or chronic obstructive pulmonary disease (COPD), caution is needed during therapy, and prior preventive actions are recommended (124). Steroid-induced side effects are common in CAR T-cell therapy such as musculoskeletal complications involving osteonecrosis and adrenal suppression. The management process includes reducing the steroid dose where possible and providing supportive care that includes physiotherapy and pain control (125). For genotoxicity and related effects, frequent blood count and genetic tests should be conducted while for treatment of the genotoxicity, the treatment depends on the severity of the effect and the type of genotoxicity. The possible side effects of corticosteroids, including hyperglycemia, are controlled by periodic modulation of corticosteroid doses and administration of insulin if required. Pancreatic side effects, though rare, need immediate intervention through the use of enzyme supplements (126) and frequent blood sugar level checks. Molecular pathways, including cytokine release syndrome, also cause nephrotic complications and are addressed in terms of fluid and electrolyte disorders. A summary containing information on the problems related to CAR-T cell therapy, conventional therapy, the pros and cons of the conventional approach, and approaches to address the challenges is presented in **Table 3**.

**Table 3:** Summary of CAR-T cell therapy, traditional treatment methods, associated challenges, and overcoming strategies.

Aspects	CAR-T cell therapy	Traditional treatment methods	Studies
**Advantages**	Long-term efficacy in some hematological malignancies (e.g., leukemia, lymphoma), and high specificity and personalized approach.	Standardized protocols for treatment, broad applicability, and immediate results in some cases	Irizarry Gatell et al. (127) Qureshi et al. (128)
**Disadvantages**	High cost of production, risk of severe CRS and neurotoxicity, tumor relapse and antigen escape, and limited availability and access	Lack of specificity, systemic toxicity, and resistance and relapse in advanced stages	An et al. (129) Brudno and Kochenderfer (4)
**Challenges**	Cytokine release syndrome (CRS), CAR-T cell persistence and manufacturing difficulties, neurotoxicity, and tumor antigen escape (tumor heterogeneity)	Radiation therapy, Chemotherapy (e.g., cytotoxic drugs), and Conventional immunotherapy (e.g., monoclonal antibodies)	An et al. (129) Irizarry Gatell et al. (127)
**Overcoming strategies**	Improvements in CAR-T design to increase efficacy and reduce side effects (e.g., armored CARs). Targeting tumor microenvironment to enhance CAR-T persistence. Use of “off the shelf” CAR-T cells. CAR-T cell engineering improvements (e.g., co-stimulatory domains).	Combination with immunotherapies to boost efficacy. Improved screening and monitoring for side effects. Dual targeting and checkpoint inhibition. Enhanced targeting to reduce antigen escape.	An et al. (129) Brudno and Kochenderfer (4) Irizarry Gatell et al. (127)

## 2 Conclusion and future prospects

Treatment for several blood-related malignancies and their associated off-target complications has been improved by CAR T-cell therapy and achieved remarkable success, especially in relapsed or refractory patients when prior therapies failed. Although CAR T-cell therapy has established a remarkable image as a promising cytotoxic therapeutic, according to our extensive data and historical review, it also induces severe toxicities such as CRS, ICANS, TLS, HLH, B-cell aplasia, GHVD, anaphylaxis, cytopenia, and coagulation disorders, and some off-target complications such as cardiovascular, musculoskeletal, pulmonary, metabolic, and pancreatic toxicities. These unavoidable toxicities are significant hurdles that patients encounter, which must be recognized early and sorted out swiftly. The implementation of standardized grading systems such as those offered by the American Society for Transplantation and Cellular Therapy (ASTCT); conventional approaches; corticosteroids; fully human generated CARs; etoposide, methotrexate, or intrathecal cytarabine; the use of hypouricemic agents; and the inhibitory CAR (iCAR) and synNotch receptor methods has been critical in improving clinical management of CAR T-cell-therapy-associated toxicities. As we learn more about the underlying mechanisms of CAR T-cell therapy-associated toxicities so will the strategies to mitigate these risks develop, leading to harmless and highly efficacious CAR T-cell treatments. Further research and progress regarding early detection, precise risk stratification, and personalized interventions for these toxicities are essential to broaden the lifesaving performance of CAR T-cell therapy to a wider pool of patients while limiting toxicity.

Despite the current limitations of CAR-T cell therapy, innovative approaches and streamlined research promise to enhance its efficacy and safety. Initially, these limitations can be tackled through careful dosing plans and innovative approaches after a clear understanding of the mechanisms underpinning them. Neurotoxicity, CRS, and cancer recurrence rates can be managed by incorporating off switches, CARs, tandem CARs, and their constructs. Gene editing innovations and artificial intelligence integration are predicted to revolutionize CAR T-cell therapy through manufacturing processes and optimizing target identification. CAR-T efficacy is expected to be boosted through the combination of therapies with immune checkpoint inhibitors and other agents that exhibit synergistic effects. Despite these innovations, its global adoption is restricted by limited availability and high cost. However, its accessibility can be widened by reducing manufacturing costs. Additionally, continuous monitoring, rapid detection, and accurate intervention with supportive care and prophylactic strategies are crucial for managing CAR T cell-associated toxicities. Overall, the future of CAR-T cell therapy has the capability of managing various toxicities provided that the costs and limitations of toxicity management are effectively addressed.

## References

[1] Sterner RC Sterner RM. CAR-T cell therapy: current limitations and potential strategies. Blood Cancer J. (2021) 11:69. doi: 10.1038/s41408-021-00459-7 33824268 PMC8024391

[2] Mohanty R Chowdhury CR Arega S Sen P Ganguly P Ganguly N. CAR T cell therapy: A new era for cancer treatment. Oncol Rep. (2019) 42:2183–95. doi: 10.3892/or.2019.7335 31578576

[3] Schubert M-L Schmitt M Wang L Ramos C Jordan K Müller-Tidow C. Side-effect management of chimeric antigen receptor (CAR) T-cell therapy. Ann Oncol. (2021) 32:34–48. doi: 10.1016/j.annonc.2020.10.478 33098993

[4] Brudno JN Kochenderfer JN. Current understanding and management of CAR T cell-associated toxicities. Nat Rev Clin Oncol. (2024) 21:1–21. doi: 10.1038/s41571-024-00903-0 38769449 PMC11529341

[5] Ghilardi G Fraietta JA Gerson JN Van Deerlin VM Morrissette JJ Caponetti GC. T cell lymphoma and secondary primary Malignancy risk after commercial CAR T cell therapy. Nat Med. (2024) 30:1–6. doi: 10.1038/s41591-024-02826-w 38266761

[6] Marker DF Kofler JK Mettenburg JA Agha ME Wiley CA. Multifocal necrotizing leukoencephalopathy with preferential microglia toxicity in a patient treated with chimeric antigen receptor T-cells and review of the literature. J Neuropathology Exp Neurol. (2020) 79:1115–21. doi: 10.1093/jnen/nlaa099 PMC7559234 32954433

[7] Locke FL Miklos DB Jacobson CA Perales M-A Kersten M-J Oluwole OO. Axicabtagene ciloleucel as second-line therapy for large B-cell lymphoma. New Engl J Med. (2022) 386:640–54. doi: 10.1056/NEJMoa2116133 34891224

[8] Neelapu SS. Managing the toxicities of CAR T-cell therapy. Hematological Oncol. (2019) 37:48–52. doi: 10.1002/hon.v37.S1 31187535

[9] Afzal A Farooque U Gillies E Hassell L. T-cell therapy-mediated myocarditis secondary to cytokine release syndrome. Cureus. (2020) 12:1–6. doi: 10.7759/cureus.10022 PMC7515743 32983717

[10] Karschnia P Jordan JT Forst DA Arrillaga-Romany IC Batchelor TT Baehring JM. Clinical presentation, management, and biomarkers of neurotoxicity after adoptive immunotherapy with CAR T cells. Blood J Am Soc Hematol. (2019) 133:2212–21. doi: 10.1182/blood-2018-12-893396 30808634

[11] Hay KA Hanafi L-A Li D Gust J Liles WC Wurfel MM. Kinetics and biomarkers of severe cytokine release syndrome after CD19 chimeric antigen receptor–modified T-cell therapy. Blood J Am Soc Hematol. (2017) 130:2295–306. doi: 10.1182/blood-2017-06-793141 PMC5701525 28924019

[12] Neelapu SS Tummala S Kebriaei P Wierda W Gutierrez C Locke FL. Chimeric antigen receptor T-cell therapy—assessment and management of toxicities. Nat Rev Clin Oncol. (2018) 15:47–62. doi: 10.1038/nrclinonc.2017.148 28925994 PMC6733403

[13] Chakraborty R Sidana S Shah GL Scordo M Hamilton BK Majhail NS. Patient-reported outcomes with chimeric antigen receptor T cell therapy: challenges and opportunities. Biol Blood Marrow Transplant. (2019) 25:e155–62. doi: 10.1016/j.bbmt.2018.11.025 PMC6511294 30500439

[14] Teachey DT Lacey SF Shaw PA Melenhorst JJ Maude SL Frey N. Identification of predictive biomarkers for cytokine release syndrome after chimeric antigen receptor T-cell therapy for acute lymphoblastic leukemia. Cancer Discovery. (2016) 6:664–79. doi: 10.1158/2159-8290.CD-16-0040 PMC5448406 27076371

[15] Lee DW Gardner R Porter DL Louis CU Ahmed N Jensen M. Current concepts in the diagnosis and management of cytokine release syndrome. Blood J Am Soc Hematol. (2014) 124:188–95. doi: 10.1182/blood-2014-05-552729 PMC4093680 24876563

[16] Park JH Rivière I Gonen M Wang X Sénéchal B Curran KJ. Long-term follow-up of CD19 CAR therapy in acute lymphoblastic leukemia. New Engl J Med. (2018) 378:449–59. doi: 10.1056/NEJMoa1709919 PMC6637939 29385376

[17] Pemmaraju N Wilson NR Senapati J Economides MP Guzman ML Neelapu SS. CD123-directed allogeneic chimeric-antigen receptor T-cell therapy (CAR-T) in blastic plasmacytoid dendritic cell neoplasm (BPDCN): Clinicopathological insights. Leukemia Res. (2022) 121:106928. doi: 10.1016/j.leukres.2022.106928 35963025

[18] Belin C Devic P Ayrignac X Dos Santos A Paix A Sirven-Villaros L. Description of neurotoxicity in a series of patients treated with CAR T-cell therapy. Sci Rep. (2020) 10:18997. doi: 10.1038/s41598-020-76055-9 33149178 PMC7642402

[19] Möhn N Bonda V Grote-Levi L Panagiota V Fröhlich T Schultze-Florey C. Neurological management and work-up of neurotoxicity associated with CAR T cell therapy. Neurological Res Pract. (2022) 4:1–10. doi: 10.1186/s42466-021-00166-5 PMC8744256 35000613

[20] Sales C Anderson MA Kuznetsova V Rosenfeld H Malpas CB Roos I. Patterns of neurotoxicity among patients receiving chimeric antigen receptor T-cell therapy: A single-centre cohort study. Eur J Neurol. (2024) 31:e16174. doi: 10.1111/ene.16174 38085272 PMC11235605

[21] Le Cacheux C Couturier A Sortais C Houot R Péré M Gastinne T. Features and outcomes of patients admitted to the ICU for chimeric antigen receptor T cell-related toxicity: a French multicentre cohort. Ann Intensive Care. (2024) 14:20–1. doi: 10.1186/s13613-024-01247-9 PMC10828176 38291184

[22] Gazeau N Liang EC Voutsinas JM Barba P Iacoboni G Kwon M. Anakinra for refractory cytokine release syndrome or immune effector cell-associated neurotoxicity syndrome after chimeric antigen receptor T cell therapy. Transplant Cell Ther. (2023) 29:430–7. doi: 10.1016/j.jtct.2023.04.001 PMC10330552 37031746

[23] Mauget M Lemercier S Quelven Q Maamar A Lhomme F De Guibert S. Impact of diagnostic investigations in the management of CAR T-cell–associated neurotoxicity. Blood Adv. (2024) 8:2491–8. doi: 10.1182/bloodadvances.2023011669 PMC11131053 38501964

[24] Nie EH Ahmadian SS Bharadwaj SN Acosta-Alvarez L Threlkeld ZD Frank MJ. Multifocal demyelinating leukoencephalopathy and oligodendroglial lineage cell loss with immune effector cell-associated neurotoxicity syndrome (ICANS) following CD19 CAR T-cell therapy for mantle cell lymphoma. J Neuropathology Exp Neurol. (2023) 82:160–8. doi: 10.1093/jnen/nlac121 PMC10655196 36592076

[25] Jacobson CA Chavez JC Sehgal AR William BM Munoz J Salles G. Axicabtagene ciloleucel in relapsed or refractory indolent non-Hodgkin lymphoma (ZUMA-5): a single-arm, multicentre, phase 2 trial. Lancet Oncol. (2022) 23:91–103. doi: 10.1016/S1470-2045(21)00591-X 34895487

[26] Abramson JS Palomba ML Gordon LI Lunning MA Wang M Arnason J. Lisocabtagene maraleucel for patients with relapsed or refractory large B-cell lymphomas (TRANSCEND NHL 001): a multicentre seamless design study. Lancet. (2020) 396:839–52. doi: 10.1016/S0140-6736(20)31366-0 32888407

[27] Shah BD Ghobadi A Oluwole OO Logan AC Boissel N Cassaday RD. KTE-X19 for relapsed or refractory adult B-cell acute lymphoblastic leukaemia: phase 2 results of the single-arm, open-label, multicentre ZUMA-3 study. Lancet. (2021) 398:491–502. doi: 10.1016/S0140-6736(21)01222-8 34097852 PMC11613962

[28] Santomasso BD Gust J Perna F. How I treat unique and difficult-to-manage cases of CAR T-cell therapy–associated neurotoxicity. Blood J Am Soc Hematol. (2023) 141:2443–51. doi: 10.1182/blood.2022017604 PMC10329188 36877916

[29] Shalabi H Harrison C Yates B Calvo KR Lee DW Shah NN. Intrathecal hydrocortisone for treatment of children and young adults with CAR T-cell immune-effector cell-associated neurotoxicity syndrome. Pediatr Blood Cancer. (2024) 71:e30741. doi: 10.1002/pbc.30741 37897136 PMC10841378

[30] Wang Y Qi K Cheng H Cao J Shi M Qiao J. Coagulation disorders after chimeric antigen receptor T cell therapy: analysis of 100 patients with relapsed and refractory hematologic Malignancies. Biol Blood Marrow Transplant. (2020) 26:865–75. doi: 10.1016/j.bbmt.2019.11.027 31786240

[31] Obeidat K Tariq MJ Gupta S. Weekend effect on TLS admission in patients with acute leukemia and lymphoma: Nationwide analysis of mortality, HD, demographics and health care utilization. Am Soc Clin Oncol. (2022) 40:e19582. doi: 10.1200/JCO.2022.40.16_suppl.e19582

[32] Moturi KR Lingamaneni P Baral B Vohra I Banskota SU Gupta S. Temporal trends in inpatient outcomes and resource utilization in patients with tumor lysis syndrome (TLS) with solid and hematologic cancers: A nationwide analysis. Am Soc Clin Oncol. (2020) 38:e19102. doi: 10.1200/JCO.2020.38.15_suppl.e19102

[33] Gangani K Fong HK Faisaluddin M Lodhi MU Manaktala P Sadolikar A. Arrhythmia in tumor lysis syndrome and associated in-hospital mortality: A nationwide inpatient analysis. J Arrhythmia. (2021) 37:121–7. doi: 10.1002/joa3.12482 PMC7896454 33664894

[34] Rios-Olais FA Gil-Lopez F Mora-Cañas A Demichelis-Gómez R. Tumor lysis syndrome is associated with worse outcomes in adult patients with acute lymphoblastic leukemia. Acta Haematologica. (2023) 147:1–11. doi: 10.1159/000534453 37963436

[35] Roy AM Konda M Goel A Meena N. 525: Characteristics And complications of tumor lysis syndrome in hematologic Malignancies. Crit Care Med. (2020) 48:243. doi: 10.1097/01.ccm.0000620444.16395.9f

[36] Adla Jala SR Gadhiya D Sakthivel H Tummala N Mal M Dhaliwal KBS. Predictors of mortality among adults with acute lymphocytic leukemia in relapse with tumor lysis syndrome during chemotherapy. J Clin Oncol. (2024) 42:e18510. doi: 10.1200/JCO.2024.42.16

[37] Feng W Jiang D Xu Y Li Y Chen L Zhao M. CDK4/6i enhances the antitumor effect of PD1 antibody by promoting TLS formation in ovarian cancer. Heliyon. (2023) 9:e19760. doi: 10.1016/j.heliyon.2023.e19760 37809574 PMC10559077

[38] Kelkar N Wang J. Clinical features and outcomes of tumor lysis syndrome in patients with gastrointestinal cancers. Am Soc Clin Oncol. (2022) 40:655–5. doi: 10.1200/JCO.2022.40.4_suppl.655

[39] Wang L Li X Zhao B Mei D Jiang J Duan J. Immune checkpoint inhibitor–associated tumor lysis syndrome: A real-world pharmacovigilance study. Front Pharmacol. (2021) 12:679207. doi: 10.3389/fphar.2021.679207 34630077 PMC8495237

[40] Bozkurt S Genc DB Vural S. Laboratory and clinical features of tumor lysis syndrome in children with non-Hodgkin lymphoma and evaluation of long-term renal functions in survivors. BMC Pediatr. (2024) 24:85. doi: 10.1186/s12887-024-04549-w 38297237 PMC10829167

[41] Ahmed AR Haque AU Amanullah F Mirza S Rahman F Muhammad S. Safety and efficacy of renal replacement therapy for acute kidney injury in tumor lysis syndrome. Asian J Pediatr Nephrol. (2020) 3:67–70. doi: 10.4103/AJPN.AJPN_17_20

[42] Cairo M Barnes Y Drea E Carrol S. MDS-065 fatalities from tumor lysis syndrome (TLS) after anti-hyperuricemic monotherapy–nationally representative, propensity score matched, retrospective study comparison of rasburicase and allopurinol. Clin Lymphoma Myeloma Leukemia. (2022) 22:S302–3. doi: 10.1016/S2152-2650(22)01393-3

[43] Monteagudo LA Boothby A Gertner E. Continuous intravenous anakinra infusion to calm the cytokine storm in macrophage activation syndrome. ACR Open Rheumatol. (2020) 2:276–82. doi: 10.1002/acr2.11135 PMC7231518 32267081

[44] Amikishiyev S Deniz R Gunver M Aghamuradov S Koca N Ince B. Pos1216 potential predictors of outcome for anakinra treatment in covid-19 patients with macrophage activation syndrome. BMJ Publishing Group Ltd. (2022) 81:937–8. doi: 10.1136/annrheumdis-2022-eular.1637

[45] Molinos-Quintana Á Alonso-Saladrigues A Herrero B Caballero-Velázquez T Galán-Gómez V Panesso M. Impact of disease burden and late loss of B cell aplasia on the risk of relapse after CD19 chimeric antigen receptor T Cell (Tisagenlecleucel) infusion in pediatric and young adult patients with relapse/refractory acute lymphoblastic leukemia: role of B-cell monitoring. Front Immunol. (2024) 14:1280580. doi: 10.3389/fimmu.2023.1280580 38292483 PMC10825008

[46] Baird JH Epstein DJ Tamaresis JS Ehlinger Z Spiegel JY Craig J. Immune reconstitution and infectious complications following axicabtagene ciloleucel therapy for large B-cell lymphoma. Blood Adv. (2021) 5:143–55. doi: 10.1182/bloodadvances.2020002732 PMC7805341 33570626

[47] Gabelli M Oporto-Espuelas M Burridge S Chu J Farish S Hedges E. Maintenance therapy for early loss of B-cell aplasia after anti-CD19 CAR T-cell therapy. Blood Adv. (2024) 8:1959–63. doi: 10.1182/bloodadvances.2023011168 PMC11021820 37820111

[48] Walton ZE Frigault MJ Maus MV. Current and emerging pharmacotherapies for cytokine release syndrome, neurotoxicity, and hemophagocytic lymphohistiocytosis-like syndrome due to CAR T cell therapy. Expert Opin Pharmacotherapy. (2024) 25:263–79. doi: 10.1080/14656566.2024.2340738 38588525

[49] Liu D Zhao J. Cytokine release syndrome: grading, modeling, and new therapy. J Hematol Oncol. (2018) 11:121. doi: 10.1186/s13045-018-0653-x 30249264 PMC6154787

[50] Nastoupil LJ Jain MD Feng L Spiegel JY Ghobadi A Lin Y. Standard-of-care axicabtagene ciloleucel for relapsed or refractory large B-cell lymphoma: results from the US Lymphoma CAR T Consortium. J Clin Oncol. (2020) 38:3119–28. doi: 10.1200/JCO.19.02104 PMC7499611 32401634

[51] Zhao W-H Liu J Wang B-Y Chen Y-X Cao X-M Yang Y. A phase 1, open-label study of LCAR-B38M, a chimeric antigen receptor T cell therapy directed against B cell maturation antigen, in patients with relapsed or refractory multiple myeloma. J Hematol Oncol. (2018) 11:1–8. doi: 10.1186/s13045-018-0681-6 30572922 PMC6302465

[52] Jain MD Smith M Shah NN. How I treat refractory CRS and ICANS after CAR T-cell therapy. Blood J Am Soc Hematol. (2023) 141:2430–42. doi: 10.1182/blood.2022017414 PMC10329191 36989488

[53] Vinnakota JM Biavasco F Schwabenland M Chhatbar C Adams RC Erny D. Immune-effector-cell-associated-neurotoxicity-syndrome (ICANS) pathophysiology is mediated by microglia TGF-β-activated kinase-1 signaling. Blood. (2023) 142:100. doi: 10.1182/blood-2023-184867

[54] Howard SC Pui C-H Ribeiro RC. Tumor lysis syndrome. Renal Dis Cancer Patients. (2014) 4:39–64. doi: 10.1016/B978-0-12-415948-8.00004-0

[55] Kozani PS Shabani S. Adverse events and Side effects of Chimeric Antigen Receptor (CAR) T cell therapy in patients with hematologic Malignancies. Trends Med Sci. (2021) 1:1–7. doi: 10.5812/tms.116301

[56] Tasian SK Gardner RA. CD19-redirected chimeric antigen receptor-modified T cells: a promising immunotherapy for children and adults with B-cell acute lymphoblastic leukemia (ALL). Ther Adv Hematol. (2015) 6:228–41. doi: 10.1177/2040620715588916 PMC4556967 26425336

[57] Grupp SA Kalos M Barrett D Aplenc R Porter DL Rheingold SR. Chimeric antigen receptor–modified T cells for acute lymphoid leukemia. New Engl J Med. (2013) 368:1509–18. doi: 10.1056/NEJMoa1215134 PMC4058440 23527958

[58] Howard SC Trifilio S Gregory TK Baxter N Mcbride A. Tumor lysis syndrome in the era of novel and targeted agents in patients with hematologic Malignancies: a systematic review. Ann Hematol. (2016) 95:563–73. doi: 10.1007/s00277-015-2585-7 26758269

[59] Brudno JN Somerville RP Shi V Rose JJ Halverson DC Fowler DH. Allogeneic T cells that express an anti-CD19 chimeric antigen receptor induce remissions of B-cell Malignancies that progress after allogeneic hematopoietic stem-cell transplantation without causing graft-versus-host disease. J Clin Oncol. (2016) 34:1112–21. doi: 10.1200/JCO.2015.64.5929 PMC4872017 26811520

[60] Tosi P Barosi G Lazzaro C Liso V Marchetti M Morra E. Consensus conference on the management of tumor lysis syndrome. haematologica. (2008) 93:1877–85. doi: 10.3324/haematol.13290 18838473

[61] Khan FY Yousef MF. Updates on the diagnosis and management of tumor lysis syndrome. Libyan Int J Oncol. (2023) 2:34–44.

[62] Ravelli A Minoia F Davì S Martini A. Macrophage activation syndrome. Handb Systemic Autoimmune Diseases. Elsevier. (2016) 11:85–106. doi: 10.1016/B978-0-444-63596-9.00004-9

[63] Neelapu SS Tummala S Kebriaei P Wierda W Locke FL Lin Y. Toxicity management after chimeric antigen receptor T cell therapy: one size does not fit'ALL'. Nat Rev Clin Oncol. (2018) 15:218–8. doi: 10.1038/nrclinonc.2018.20 PMC6716606 29434334

[64] Ceppi F summers C Gardner RA. Hematologic and non-CRS toxicities. In: Chimeric Antigen Receptor T-Cell Therapies for Cancer. Radarweg 29, 1043 NX Amsterdam, Netherlands: Elsevier (2020). p. 107–12.

[65] Hashmi H Bachmeier C Chavez JC Song J Hussaini M Krivenko G. Haemophagocytic lymphohistiocytosis has variable time to onset following CD19 chimeric antigen receptor T cell therapy. Br J Haematology. (2019) 187:e35–8. doi: 10.1111/bjh.v187.2 31410842

[66] Maude SL Barrett D Teachey DT Grupp SA. Managing cytokine release syndrome associated with novel T cell-engaging therapies. Cancer J. (2014) 20:119–22. doi: 10.1097/PPO.0000000000000035 PMC4119809 24667956

[67] Arnold DE Maude SL Callahan CA Dinofia AM Grupp SA Heimall JR. Subcutaneous immunoglobulin replacement following CD19-specific chimeric antigen receptor T-cell therapy for B-cell acute lymphoblastic leukemia in pediatric patients. Pediatr Blood Cancer. (2020) 67:e28092. doi: 10.1002/pbc.28092 31793170

[68] Maude SL Laetsch TW Buechner J Rives S Boyer M Bittencourt H. Tisagenlecleucel in children and young adults with B-cell lymphoblastic leukemia. New Engl J Med. (2018) 378:439–48. doi: 10.1056/NEJMoa1709866 PMC5996391 29385370

[69] Mueller KT Waldron E Grupp SA Levine JE Laetsch TW Pulsipher MA. Clinical pharmacology of tisagenlecleucel in B-cell acute lymphoblastic leukemia. Clin Cancer Res. (2018) 24:6175–84. doi: 10.1158/1078-0432.CCR-18-0758 PMC7433345 30190371

[70] Lee DW Kochenderfer JN Stetler-Stevenson M Cui YK Delbrook C Feldman SA. T cells expressing CD19 chimeric antigen receptors for acute lymphoblastic leukaemia in children and young adults: a phase 1 dose-escalation trial. Lancet. (2015) 385:517–28. doi: 10.1016/S0140-6736(14)61403-3 PMC7065359 25319501

[71] Schuster SJ Bishop MR Tam CS Waller EK Borchmann P Mcguirk JP. Tisagenlecleucel in adult relapsed or refractory diffuse large B-cell lymphoma. New Engl J Med. (2019) 380:45–56. doi: 10.1056/NEJMoa1804980 30501490

[72] Awasthi R Pacaud L Waldron E Tam CS Jäger U Borchmann P. Tisagenlecleucel cellular kinetics, dose, and immunogenicity in relation to clinical factors in relapsed/refractory DLBCL. Blood Adv. (2020) 4:560–72. doi: 10.1182/bloodadvances.2019000525 PMC7013261 32045475

[73] Yakoub-Agha I Chabannon C Bader P Basak GW Bonig H Ciceri F. Management of adults and children undergoing chimeric antigen receptor T-cell therapy: best practice recommendations of the European Society for Blood and Marrow Transplantation (EBMT) and the Joint Accreditation Committee of ISCT and EBMT (JACIE). Haematologica. (2020) 105:297. doi: 10.3324/haematol.2019.229781 31753925 PMC7012497

[74] Logue JM Zucchetti E Bachmeier CA Krivenko GS Larson V Ninh D. Immune reconstitution and associated infections following axicabtagene ciloleucel in relapsed or refractory large B-cell lymphoma. Haematologica. (2021) 106:978. doi: 10.3324/haematol.2019.238634 32327504 PMC8017820

[75] Hill JA Krantz EM Hay KA Dasgupta S Stevens-Ayers T Bender Ignacio RA. Durable preservation of antiviral antibodies after CD19-directed chimeric antigen receptor T-cell immunotherapy. Blood Adv. (2019) 3:3590–601. doi: 10.1182/bloodadvances.2019000717 PMC6880890 31743392

[76] Maus MV Haas AR Beatty GL Albelda SM Levine BL Liu X. T cells expressing chimeric antigen receptors can cause anaphylaxis in humans. Cancer Immunol Res. (2013) 1:26–31. doi: 10.1158/2326-6066.CIR-13-0006 24777247 PMC3888798

[77] Brudno JN Lam N Vanasse D Shen Y-W Rose JJ Rossi J. Safety and feasibility of anti-CD19 CAR T cells with fully human binding domains in patients with B-cell lymphoma. Nat Med. (2020) 26:270–80. doi: 10.1038/s41591-019-0737-3 PMC7781235 31959992

[78] Gelis S Verdesoto J-T Pascal M Muñoz-Cano RM. Hypersensitivity reactions to monoclonal antibodies: new approaches. Curr Treat Options Allergy. (2022) 9:394–408. doi: 10.1007/s40521-022-00318-1

[79] Passey C Suryawanshi S Sanghavi K Gupta M. Reporting, visualization, and modeling of immunogenicity data to assess its impact on pharmacokinetics, efficacy, and safety of monoclonal antibodies. AAPS J. (2018) 20:1–13. doi: 10.1208/s12248-018-0194-9 29484520

[80] Rosenberg AS Sauna ZE. Immunogenicity assessment during the development of protein therapeutics. J Pharm Pharmacol. (2018) 70:584–94. doi: 10.1111/jphp.12810 28872677

[81] Zhou Y Penny HL Kroenke MA Bautista B Hainline K Chea LS. Immunogenicity assessment of bispecific antibody-based immunotherapy in oncology. J Immunotherapy Cancer. (2022) 10:e004225. doi: 10.1136/jitc-2021-004225 PMC9024276 35444060

[82] Asghar MS Shah SMI Rani A Kazmi S Savul IS Ukrani J. Toxicities of CAR T-cell therapy: a review of current literature. Ann Med Surg. (2023) 85:6013–20. doi: 10.1097/MS9.0000000000001375 PMC10718333 38098580

[83] Ferrara JL Levine JE Reddy P Holler E. Graft-versus-host disease. Lancet. (2009) 373:1550–61. doi: 10.1016/S0140-6736(09)60237-3 PMC2735047 19282026

[84] Turtle CJ Hanafi L-A Berger C Gooley TA Cherian S Hudecek M. CD19 CAR–T cells of defined CD4+: CD8+ composition in adult B cell ALL patients. J Clin Invest. (2016) 126:2123–38. doi: 10.1172/JCI85309 PMC4887159 27111235

[85] Lai X Sun YY Chang LJ Ma YR Gu XZ Yao XM. Could cytokine release syndrome induce acute myelofibrosis in CD19 chimeric antigen receptor T cells therapy? Bioengineered. (2020) 11:824–8. doi: 10.1080/21655979.2020.1791597 PMC8291836 32772769

[86] Iurlo A Cattaneo D Bucelli C. Management of myelofibrosis: from diagnosis to new target therapies. Curr Treat Options Oncol. (2020) 21:1–14. doi: 10.1007/s11864-020-00734-y 32350623

[87] Kuykendall AT. Updates in the management of myelofibrosis. J Natl Compr Cancer Network. (2023) 21:23–6. doi: 10.6004/jnccn.2023.5038

[88] Cappell KM Kochenderfer JN. Long-term outcomes following CAR T cell therapy: what we know so far. Nat Rev Clin Oncol. (2023) 20:359–71. doi: 10.1038/s41571-023-00754-1 PMC10100620 37055515

[89] Martin T Usmani SZ Berdeja JG Agha M Cohen AD Hari P. Ciltacabtagene autoleucel, an anti–B-cell maturation antigen chimeric antigen receptor T-cell therapy, for relapsed/refractory multiple myeloma: CARTITUDE-1 2-year follow-up. J Clin Oncol. (2023) 41:1265–74. doi: 10.1200/JCO.22.00842 PMC9937098 35658469

[90] Brudno JN Natrakul D Lam N Dulau-Florea A Yuan CM Kochenderfer JN. Acute and delayed cytopenias following CAR T-cell therapy: an investigation of risk factors and mechanisms. Leukemia Lymphoma. (2022) 63:1849–60. doi: 10.1080/10428194.2022.2056172 PMC11627212 35389319

[91] Strati P Varma A Adkins S Nastoupil LJ Westin JR Hagemeister FB. Hematopoietic recovery and immune reconstitution after axicabtagene ciloleucel in patients with large B-cell lymphoma. Haematologica. (2021) 106:2667. doi: 10.3324/haematol.2020.254045 32732355 PMC8485681

[92] Cappell KM Sherry RM Yang JC Goff SL Vanasse DA Mcintyre L. Long-term follow-up of anti-CD19 chimeric antigen receptor T-cell therapy. J Clin Oncol. (2020) 38:3805–15. doi: 10.1200/JCO.20.01467 PMC7655016 33021872

[93] Cordeiro A Bezerra ED Hirayama AV Hill JA Wu QV Voutsinas J. Late events after treatment with CD19-targeted chimeric antigen receptor modified T cells. Biol Blood Marrow Transplant. (2020) 26:26–33. doi: 10.1016/j.bbmt.2019.08.003 31419568 PMC6953906

[94] Jain T Knezevic A Pennisi M Chen Y Ruiz JD Purdon TJ. Hematopoietic recovery in patients receiving chimeric antigen receptor T-cell therapy for hematologic Malignancies. Blood Adv. (2020) 4:3776–87. doi: 10.1182/bloodadvances.2020002509 PMC7422135 32780846

[95] Locke FL Ghobadi A Jacobson CA Miklos DB Lekakis LJ Oluwole OO. Long-term safety and activity of axicabtagene ciloleucel in refractory large B-cell lymphoma (ZUMA-1): a single-arm, multicentre, phase 1–2 trial. Lancet Oncol. (2019) 20:31–42. doi: 10.1016/S1470-2045(18)30864-7 30518502 PMC6733402

[96] Schubert M-L Dietrich S Stilgenbauer S Schmitt A Pavel P Kunz A. Feasibility and safety of CD19 chimeric antigen receptor T cell treatment for B cell lymphoma relapse after allogeneic hematopoietic stem cell transplantation. Biol Blood Marrow Transplant. (2020) 26:1575–80. doi: 10.1016/j.bbmt.2020.04.025 32422254

[97] Fried S Avigdor A Bielorai B Meir A Besser MJ Schachter J. Early and late hematologic toxicity following CD19 CAR-T cells. Bone marrow Transplant. (2019) 54:1643–50. doi: 10.1038/s41409-019-0487-3 30809033

[98] Jain MD Davila ML. Concise review: emerging principles from the clinical application of chimeric antigen receptor T cell therapies for B cell Malignancies. Stem Cells. (2018) 36:36–44. doi: 10.1002/stem.2715 29024301

[99] Kansagra AJ Frey NV Bar M Laetsch TW Carpenter PA Savani BN. Clinical utilization of chimeric antigen receptor T cells in B cell acute lymphoblastic leukemia: an expert opinion from the European Society for Blood and Marrow Transplantation and the American Society for Transplantation and Cellular Therapy. Biol Blood Marrow Transplant. (2019) 25:e76–85. doi: 10.1016/j.bbmt.2018.12.068 PMC8335749 30576834

[100] Mahmoudjafari Z Hawks KG Hsieh AA Plesca D Gatwood KS Culos KA. American Society for Blood and Marrow Transplantation Pharmacy Special Interest Group survey on chimeric antigen receptor T cell therapy administrative, logistic, and toxicity management practices in the United States. Biol Blood Marrow Transplant. (2019) 25:26–33. doi: 10.1016/j.bbmt.2018.09.024 30266675

[101] Lin Q Liu X Han L Liu L Fang B Gao Q. Autologous hematopoietic stem cell infusion for sustained myelosuppression after BCMA–CAR-T therapy in patient with relapsed myeloma. Bone Marrow Transplant. (2020) 55:1203–5. doi: 10.1038/s41409-019-0674-2 PMC7269899 31537902

[102] Xu J Chen L-J Yang S-S Sun Y Wu W Liu Y-F. Exploratory trial of a biepitopic CAR T-targeting B cell maturation antigen in relapsed/refractory multiple myeloma. Proc Natl Acad Sci. (2019) 116:9543–51. doi: 10.1073/pnas.1819745116 PMC6510991 30988175

[103] Miao L Zhang Z Ren Z Li Y. Reactions related to CAR-T cell therapy. Front Immunol. (2021) 12:663201. doi: 10.3389/fimmu.2021.663201 33995389 PMC8113953

[104] Jiang H Liu L Guo T Wu Y Ai L Deng J. Improving the safety of CAR-T cell therapy by controlling CRS-related coagulopathy. Ann Hematol. (2019) 98:1721–32. doi: 10.1007/s00277-019-03685-z 31055613

[105] Helms J Merdji H Loewert S Severac F Monnier A Kaurin J. Disseminated intravascular coagulation is strongly associated with severe acute kidney injury in patients with septic shock. Ann Intensive Care. (2023) 13(1):119. doi: 10.1186/s13613-023-01216-8 38038826 PMC10692023

[106] Triplett DA. Coagulation and bleeding disorders: review and update. Clin Chem. (2000) 46:1260–9. doi: 10.1093/clinchem/46.8.1260 10926920

[107] Morgan RA Yang JC Kitano M Dudley ME Laurencot CM Rosenberg SA. Case report of a serious adverse event following the administration of T cells transduced with a chimeric antigen receptor recognizing ERBB2. Mol Ther. (2010) 18:843–51. doi: 10.1038/mt.2010.24 PMC2862534 20179677

[108] Roybal KT Rupp LJ Morsut L Walker WJ Mcnally KA Park JS. Precision tumor recognition by T cells with combinatorial antigen-sensing circuits. Cell. (2016) 164:770–9. doi: 10.1016/j.cell.2016.01.011 PMC4752902 26830879

[109] Penack O Koenecke C. Complications after CD19+ CAR T-cell therapy. Cancers. (2020) 12:3445. doi: 10.3390/cancers12113445 33228221 PMC7699604

[110] Alvi RM Frigault MJ Fradley MG Jain MD Mahmood SS Awadalla M. Cardiovascular events among adults treated with chimeric antigen receptor T-cells (CAR-T). J Am Coll Cardiol. (2019) 74:3099–108. doi: 10.1016/j.jacc.2019.10.038 PMC6938409 31856966

[111] Moriyama S Fukata M Yokoyama T Ueno S Nunomura T Mori Y. Case report: cardiac tamponade in association with cytokine release syndrome following CAR-T cell therapy. Front Cardiovasc Med. (2022) 9:848091. doi: 10.3389/fcvm.2022.848091 35387436 PMC8977736

[112] Sarfati S Norbert ME Hérault A Giry M Makké J Grall M. Case report: CAR-T cell therapy-induced cardiac tamponade. Front Cardiovasc Med. (2023) 10:1132503. doi: 10.3389/fcvm.2023.1132503 37020516 PMC10067676

[113] Cao Y Liu Y Zhang R Zhai W Ma Q Wei J. Cardiac involvement in a patient with B-cell lymphoblastic lymphoma/acute lymphoblastic leukemia and a history of allogeneic hematopoietic stem cell transplantation and CAR T-cell therapy: A case report. Front Immunol. (2023) 13:1052336. doi: 10.3389/fimmu.2022.1052336 36685607 PMC9849371

[114] Tao JJ Roszkowska N Majure DT Mahmood SS. Coronary vasospasm during infusion of CD-19 directed chimeric antigen receptor T-cell therapy: a case report. Eur Heart Journal-Case Rep. (2023) 7:ytad342. doi: 10.1093/ehjcr/ytad342 PMC10398419 37547374

[115] Elmarasi M Elkonaissi I Elsabagh AA Elsayed E Elsayed A Elsayed B. CAR-T cell therapy: Efficacy in management of cancers, adverse effects, dose-limiting toxicities and long-term follow up. Int Immunopharmacol. (2024) 135:112312. doi: 10.1016/j.intimp.2024.112312 38788449

[116] Salem J-E Ederhy S Lebrun-Vignes B Moslehi JJ. Cardiac events associated with chimeric antigen receptor T-cells (CAR-T) A vigiBase perspective. J Am Coll Cardiol. (2020) 75:2521–3. doi: 10.1016/j.jacc.2020.02.070 32408984

[117] Lefebvre B Kang Y Smith AM Frey NV Carver JR Scherrer-Crosbie M. Cardiovascular effects of CAR T cell therapy: a retrospective study. Cardio Oncol. (2020) 2:193–203. doi: 10.1016/j.jaccao.2020.04.012 PMC7413146 32776016

[118] Wudhikarn K Pennisi M Garcia-Recio M Flynn JR Afuye A Silverberg ML. DLBCL patients treated with CD19 CAR T cells experience a high burden of organ toxicities but low nonrelapse mortality. Blood Adv. (2020) 4:3024–33. doi: 10.1182/bloodadvances.2020001972 PMC7362382 32614964

[119] Haas AR Golden RJ Litzky LA Engels B Zhao L Xu F. Two cases of severe pulmonary toxicity from highly active mesothelin-directed CAR T cells. Mol Ther. (2023) 31:2309–25. doi: 10.1016/j.ymthe.2023.06.006 PMC10422001 37312454

[120] Fitzgerald JC Weiss SL Maude SL Barrett DM Lacey SF Melenhorst JJ. Cytokine release syndrome after chimeric antigen receptor T cell therapy for acute lymphoblastic leukemia. Crit Care Med. (2017) 45:e124–31. doi: 10.1097/CCM.0000000000002053 PMC5452983 27632680

[121] Astrakhan A Sather BD Ryu BY Khim S Singh S Humblet-Baron S. Ubiquitous high-level gene expression in hematopoietic lineages provides effective lentiviral gene therapy of murine Wiskott-Aldrich syndrome. Blood J Am Soc Hematol. (2012) 119:4395–407. doi: 10.1182/blood-2011-03-340711 PMC3362358 22431569

[122] Cornetta K Duffy L Turtle CJ Jensen M Forman S Binder-Scholl G. Absence of replication-competent lentivirus in the clinic: analysis of infused T cell products. Mol Ther. (2018) 26:280–8. doi: 10.1016/j.ymthe.2017.09.008 PMC5762981 28970045

[123] Ruella M Xu J Barrett DM Fraietta JA Reich TJ Ambrose DE. Induction of resistance to chimeric antigen receptor T cell therapy by transduction of a single leukemic B cell. Nat Med. (2018) 24:1499–503. doi: 10.1038/s41591-018-0201-9 PMC6511988 30275568

[124] Gutierrez C Neilan TG Grover NS. How I approach optimization of patients at risk of cardiac and pulmonary complications after CAR T-cell therapy. Blood J Am Soc Hematol. (2023) 141:2452–9. doi: 10.1182/blood.2022017579 PMC10329189 36827628

[125] Liu D Ahmet A Ward L Krishnamoorthy P Mandelcorn ED Leigh R. A practical guide to the monitoring and management of the complications of systemic corticosteroid therapy. Allergy Asthma Clin Immunol. (2013) 9:1–25. doi: 10.1186/1710-1492-9-30 23947590 PMC3765115

[126] Brudno JN Kochenderfer JN. Toxicities of chimeric antigen receptor T cells: recognition and management. Blood J Am Soc Hematol. (2016) 127:3321–30. doi: 10.1182/blood-2016-04-703751 PMC4929924 27207799

[127] Irizarry Gatell VM Huang J Puglianini OAC. CAR T-cell therapy. Anesth Oncological Surgery. Springer. (2024) 5:35–44. doi: 10.1007/978-3-031-50977-3_5

[128] Qureshi Z Altaf F Jamil A Siddique R. Optimization strategies in CAR T-cell therapy: A comprehensive evaluation of cytopenia, HLH/MAS, and other adverse events. Am J Clin Oncol. (2024) 47:607–15. doi: 10.1097/COC.0000000000001124 38907604

[129] An J Zhao J Zou P Zhang Y Wei J Tian W. Infections associated with CAR-T cell therapy in patients with relapsed refractory multiple myeloma: Risks and prevention strategies. Cancer Med. (2024) 13:e7372. doi: 10.1002/cam4.v13.12 38923216 PMC11196838

